# Blended police firearms training improves performance in shoot/don't shoot scenarios: a systematic replication with police cadets

**DOI:** 10.3389/fpsyg.2024.1495812

**Published:** 2024-12-18

**Authors:** Joshua Olma, Christine Sutter, Sandra Sülzenbrück

**Affiliations:** ^1^Institute of Traffic and Engineering Psychology, German Police University, Münster, Germany; ^2^Department of Business Studies, Westfälische Hochschule, Gelsenkirchen, Germany

**Keywords:** police, vision, attention, training, decision-making, shooting, experience, expertise

## Abstract

Senior police officers' tactical gaze control and visual attention improve with an individual video-based police firearms training. To validate the efficacy of said intervention training, a previous experiment was systematically replicated with a sample of *N* = 52 second-year police cadets. Participants were randomly assigned to the intervention training that focused on situational awareness, tactical gaze control, and visual attention, or an active control training that addressed traditional marksmanship skills. In a pre- and post-test, they had to engage in dynamic shoot/don't shoot video scenarios in an indoor firing range. Overall, the previous findings were replicated: Baseline levels of performance were elevated, yet the intervention group significantly improved their response time and time until the first hit. False positive decision-making cannot be reported at all; false negatives were marginal in the pre-test and eliminated after training. Further, the outcomes of the previous sample of senior officers and the present sample of cadets are compared and lead to the conclusion that the presented approach is a valuable extension of current training standards for both senior police officers and police cadets.

## 1 Introduction

Police work is inherently dangerous due to the unpredictable nature of situations police officers face daily. They encounter potentially violent individuals and hazardous environments, all of which pose significant risks to their physical safety. Hence, police officers must demonstrate high proficiency in accurate threat assessment and, moreover, adequate reaction (cf. Helsen and Starkes, 1999; Martaindale, 2020; Vickers and Lewinski, 2012). Recent research (Heusler, 2023; Heusler and Sutter, 2020; Olma et al., 2024) emphasizes that targeted training of visual perception is a key element of police work. Successful threat-detection is hardly possible without a visual search for suspicious and potentially hazardous objects. Apparently, years of service are relevant; experienced police officers prove superior to less experienced or non-police officers in terms of gaze patterns, reaction times, marksmanship, threat-detection, and orientation toward threats (Alexander et al., 2024; Helsen and Starkes, 1999; Heusler and Sutter, 2022a; Nieuwenhuys and Oudejans, 2011; Vickers and Lewinski, 2012). Although the exception, police use of firearms is a crucial skill that goes beyond traditional marksmanship. While there is often only a fraction of a second to decide whether to shoot or not, this decision must be based on the best possible assessment of the situation. For this assessment, police officers must know what to expect (situational awareness), what to focus on (tactical gaze control and visual attention), and how to guide their decisions (decision-making). The trigger is only pulled at the very end of this sensorimotor cascade for which visual perception is a major facilitator (cf. Sack and Sutter, 2017; Sutter et al., 2013). Given the premise that good decision-making relies on even better visual information processing, it seems feasible to develop concepts in order to optimally prepare police officers for such extraordinary situations. To this end, numerous training approaches have been examined over the past decades (for an overview, see Olma et al., 2024), which differed in many aspects: Some used live ammunition, others resorted to lasers or FX ammunition. While some relied on interactive role-playing, others focused on computer-based simulations or video scenarios. Overall, two insights are worth noting: First, just one or a few training sessions are sufficient to improve perceptual performance and tactical gaze control (Helsen and Starkes, 1999; Heusler and Sutter, 2022b; Nieuwenhuys and Oudejans, 2011; Olma et al., 2024). Second, task-oriented cognitive training is beneficial to participants' shoot/don't shoot performance (Biggs et al., 2015, 2021; Biggs and Pettijohn, 2021; Hamilton et al., 2019; Heusler and Sutter, 2022b; McCraty and Atkinson, 2012; Olma et al., 2024; Preddy et al., 2019). Nevertheless, studies show that the transfer from research to practice is subject to major challenges (cf. Kleygrewe et al., 2022; Staller et al., 2022, 2023): The decontextualized nature of training tasks often isolates police firearms skills from practical application, limiting their operational relevance. Traditional, linear training models often disregard didactical principles and lack the integration needed to transfer knowledge and skills effectively to dynamic environments. Oversimplification of complex tasks ultimately leads to a mismatch between the learners' abilities and situational demands. Resource constraints, including limited training facilities and high learner-to-trainer ratios, further impede the delivery of effective and learner-centered training.

In their field study, Heusler and Sutter (2022b) designed an intervention training that laid the foundation for addressing these challenges: They gave one session of frontal classroom teaching accompanied by practical shooting tasks for application to a sample of police cadets. In the light of previous literature (cf. Biggs et al., 2015; Hamilton et al., 2019; Nieuwenhuys and Oudejans, 2011; Taylor, 2020; Vickers and Lewinski, 2012), the authors focused on the perceptual aspects of shooting that precede good decision-making; situational awareness, tactical gaze control, and visual attention. In the intervention group, participants were trained to optimize their perception of visual information by keeping a low muzzle position and both eyes open. As a consequence of optimized information processing, decision-making should be facilitated, and response time and time until the first effective hit should decrease. In practical shooting tasks, participants directly applied the theoretical input. In a pre-post setting, the effectiveness of the intervention training was evaluated against an active control group receiving a traditional firearms training that focused on marksmanship skills. In dynamic shoot/don't shoot scenarios, the intervention group improved their muzzle position, decision making, and response time to a greater extent than the active control group.

Building on this template, Olma et al. (2024) embedded the training into a didactical framework aligned with the *Four-Component Instructional Design Model* (4C/ID; for a detailed description, see van Merrienboer, 2020). Focusing on the development of complex skills and increased transfer of learning, the 4C/ID integrates the principles of the renowned *Cognitive Load Theory* (CLT; Sweller, 1994; Sweller et al., 1998): It claims that four components (*learning tasks, supportive information, procedural information*, and *part-task practice*) are necessary to balance cognitive load across the learning process and to enable skill acquisition and learning of complex tasks. *Learning tasks* integrate non-routine and routine skills by providing an authentic and variable task experience. They are divided into manageable levels, and while complexity is gradually increased, support diminishes (scaffolding) which corresponds to the CLT's emphasis on managing intrinsic cognitive load. Olma et al. (2024) had their participants engage in exercises that gradually increased in task fidelity and difficulty while demanding routine skills (e.g., basic marksmanship skills) and non-routine aspects (e.g., inhibitory control and active decision-making). *Supportive information* facilitates the learning and performance of non-routine aspects by providing classic “theory” on how to approach task-related problems. In contrast, *procedural information* facilitates the learning and performance of routine aspects by providing feedback and step-by-step instruction just in time. Providing supportive and procedural information aligns with the CLT's requirement of avoiding extraneous cognitive load. Regarding the supportive information, Olma et al. (2024) switched from frontal classroom teaching to a blended and individual video-based teaching format: Participants were presented with a self-produced educational video that delivered the theoretical input while the practical training was carefully aligned with the principles of the 4C/ID. Not only did the blended teaching format enhance the transfer from theory to practice, but it maximized the standardization of the training. Procedural information was given with the intention of not only emphasizing the errors, but also explaining the causes and potential solutions. *Part-task practice* enables high levels of automaticity by repetitive, additional practice. Olma et al. (2024) omitted the part-task practice because their learning tasks provided a sufficient amount of practice (cf. van Merrienboer, 2020). Adherence to precise design principles of the 4C/ID enhances the instructional material and increases germane cognitive load, i.e., the mental effort devoted to processing and understanding the material, which CLT suggests should be maximized to foster learning. The participants were not given live ammunition but a gas-operated replica that barely differed from their service weapon. The advantages over live ammunition were safety aspects and economic considerations. Olma et al. (2024) assessed the efficacy of the intervention training with experienced senior police officers. Opposed to the results of Heusler and Sutter (2022b), their sample already demonstrated a high level of tactical gaze control and decision-making accuracy in the pre-test, which is why neither group could improve the latter two. In the post-test, however, the intervention group took less time to respond and engage in effective fire than the control group. Consequently, Olma et al. (2024) deduced that even experienced senior police officers seem susceptible to training, although absolute training effects may be stronger for cadets.

Based on the findings of Heusler and Sutter (2022b) and Olma et al. (2024), it is reasonable to assume that cadets internalize new training content even more than experienced professionals who may learn some new aspects or refresh their knowledge. Many years of service and the associated experience may give senior police officers a head start in terms of prior knowledge, problem-solving strategies, and efficient management of the learning process (Bransford and Cocking, 2000; Kalyuga, 2011; Sweller, 2011). However, their asset might come as an obstacle when they gain new knowledge that contradicts their existing knowledge and beliefs: Studies suggest that rigid knowledge structures make it difficult to integrate new knowledge, change mental models, or objectively evaluate contradictory information (Chinn and Brewer, 1993; Feltovich et al., 1997; Nickerson, 1998). Yet, research on the differences in learning processes between novices and experts in shooting or (police) firearms training is scarce. This might also be due to the synonymously but inaccurate use of the terms *experience* and *expertise* (Bransford and Cocking, 2000; Chi et al., 1988): Experience refers to the accumulation of knowledge or skill over time that is primarily quantitative and does not necessarily result in elevated levels of performance. Expertise refers to the high level of skill or knowledge in a particular domain that is rather qualitative and results from deliberate practice and reflective learning. Educational programs for teaching complex skills or professional competencies, such as the 4C/ID (van Merrienboer, 2020), are indispensable support to achieve expertise. In the context of police use of firearms, expertise is achieved through targeted training. Naturally, police cadets are only developing their expertise and therefore have little compared to senior police officers who completed their training and maintain a relatively constant level of expertise (mostly special forces can successively increase their expertise through regular exercises and further education). In contrary, experience is hereafter defined by the number of years of service. Police cadets have none while experience levels of senior police officers can vary; the mean in the previous study was 13 years of service (Olma et al., 2024). Nonetheless, the intertwined association between experience and expertise prompted the question of whether inexperienced police cadets would benefit from individual video-based intervention training to a different extent than the senior police officers. Hence, the previous study was systematically replicated to validate the intervention training's efficacy on shoot/don't shoot performance in a pre-registered lab experiment; the only deviation was the new sample of police cadets. In line with the previous studies (Heusler and Sutter, 2022b; Olma et al., 2024), the following hypotheses were examined:

- Maintaining a low muzzle position will allow an unrestricted view on the suspect's movements and facilitate the perception of visual information. Post-training, the intervention group will bring their handgun to eyesight level later than the control group (hypothesis 1).
- Keeping both eyes open will allow a wider field of vision and optimize the perception of visual information. Post-training, the intervention group will keep their eyes open longer right before shooting than the control group (hypothesis 2).
- An optimized visual information processing and less uncertainty of the suspect's movements will reduce the proportion of false negative decisions (shoot scenarios) and false positive decisions (don't shoot scenarios). Post-training, the intervention group will improve their number of correct decisions to shoot in shoot scenarios (correct positive) and to not shoot in don't shoot scenarios (correct negative) to a greater extent than the control group (hypothesis 3).
- More accurate decision-making and optimized perception of visual information will also allow decreased response times. Post-training, the intervention group will shoot faster than the control group (hypothesis 4).
- Consequently, decreased response times will allow decreased times until an effective hit. Post-training, the intervention group will hit the attacker faster than the control group (hypothesis 5).
- As a manipulation check, the control group's focus on speed and accuracy will allow a better performance in traditional marksmanship skills. Post-training, the control group will improve their traditional marksmanship skills more than the intervention group (hypothesis 6).

## 2 Materials and methods

### 2.1 Participants

In cooperation with the Hessian Police College (“*Hessische Hochschule für öffentliches Management und Sicherheit*”), *N* = 52 second-year police cadets (female = 21; male = 31) with a mean age of 23 years (*SD* = 3.20, *min*. = 20, *max*. = 34) volunteered for the study. On average, the participants had taken 93.35 firearms lessons (*SD* = 12.90) prior to the experiment, and on a scale from 1 (none) to 5 (very high), they rated their proficiency with their service weapon at 3.77 (*SD* = 0.55). All police cadets participated voluntarily and could abort the study at any time. They gave their informed consent for inclusion before participating in the study and were debriefed at the end. The sample had normal or corrected to normal vision (two participants wore contact lenses; no prescribed glasses).

A total of *n* = 25 participants was randomly assigned to the control group, whereas *n* = 27 participants received the intervention training. The sample size was in line with previous studies (Heusler and Sutter, 2022b; Olma et al., 2024). To rule out bias, the participants' *Gender, Age, Lessons*, and *Proficiency* were treated as possible confounding variables and potential differences in group characteristics were statistically analyzed: Both groups did not differ with regard to gender distribution [control group: female = 9, male = 16; intervention group: female = 12, male = 15; $\chi_{\left(1,\text{N}=52\right)}^{2}$ = 0.11, *p* = 0.736] or mean age (mean age control group = 23.36, *SD* = 3.46; mean age intervention group = 23.00, *SD* = 2.99; *W* = 337.50, *p* = 1.00). No significant differences were observed in terms of mean lessons [mean lessons control group = 92.00, *SD* = 10.10; mean lessons intervention group = 94.59, *SD* = 15.12; *t*_(50)_ = −0.72, *p* = 0.474] and mean proficiency [mean proficiency control group = 3.76, *SD* = 0.60; mean proficiency intervention group = 3.78, *SD* = 0.51; *t*_(50)_ = −0.12, *p* = 0.908]. Since both groups did not differ significantly in the expression of *Gender, Age, Lessons*, and *Proficiency*, it was assumed that the results were not biased by those characteristics.

### 2.2 Pre- and post-test

An indoor firing range was set up in an empty classroom (20.00 meters by 8.00 meters) at the Hessian Police College. With an aluminum groove profile, a stable bracket was constructed to which a white paper canvas (2.00 meters width by 11.00 meters length, unrolled to a height of 2.20 meters) was attached. The area behind the bracket was lined with a white sheet and served as a bullet trap. The shooting distance was marked on the ground at 4.50 meters from the paper canvas. To facilitate *post-hoc* analysis, a night vision camera (TP-Link Tapo C200) was placed behind the paper canvas to capture the shooting performance precisely despite dim lighting. An Epson 2247U projector (video and sound presentation) served for the target presentation and was controlled by an experimental computer (Dell Latitude 5410). For eye-tracking, the mobile and calibration-free eye-tracking system “Neon” by Pupil Labs was used. It allowed for eyelid recording (192 × 192 pixels at 200 Hz) and first-person recording (1,600 × 1,200 pixels at 30 Hz), including sound. The device was connected to a Motorola Edge 40 Pro smartphone, which the participants could conveniently store in any pocket. During the pre- and post-test, the Neon glasses served as eye protection. For shooting, participants were given a gas-operated replica of a Heckler and Koch SFP9 handgun (in some countries: Heckler and Koch VP9). Since this handgun is almost identical to the actual service weapon in the sample (Heckler and Koch P30), the participants experienced little difficulty handling the weapon and it fit into their service holster. The handgun operates on small airsoft bullets, which are powered by a gas tank in the magazine. This also allows semi-automatic use of the handgun, including recoil and bolt catch, once the magazine is shot empty. The handgun cannot be fired with an empty gas tank or with no magazine inserted. In the safety instruction, the participants were briefed on the correct handling of this economic and non-lethal weapon prior to the first task.

**Figure 1:**
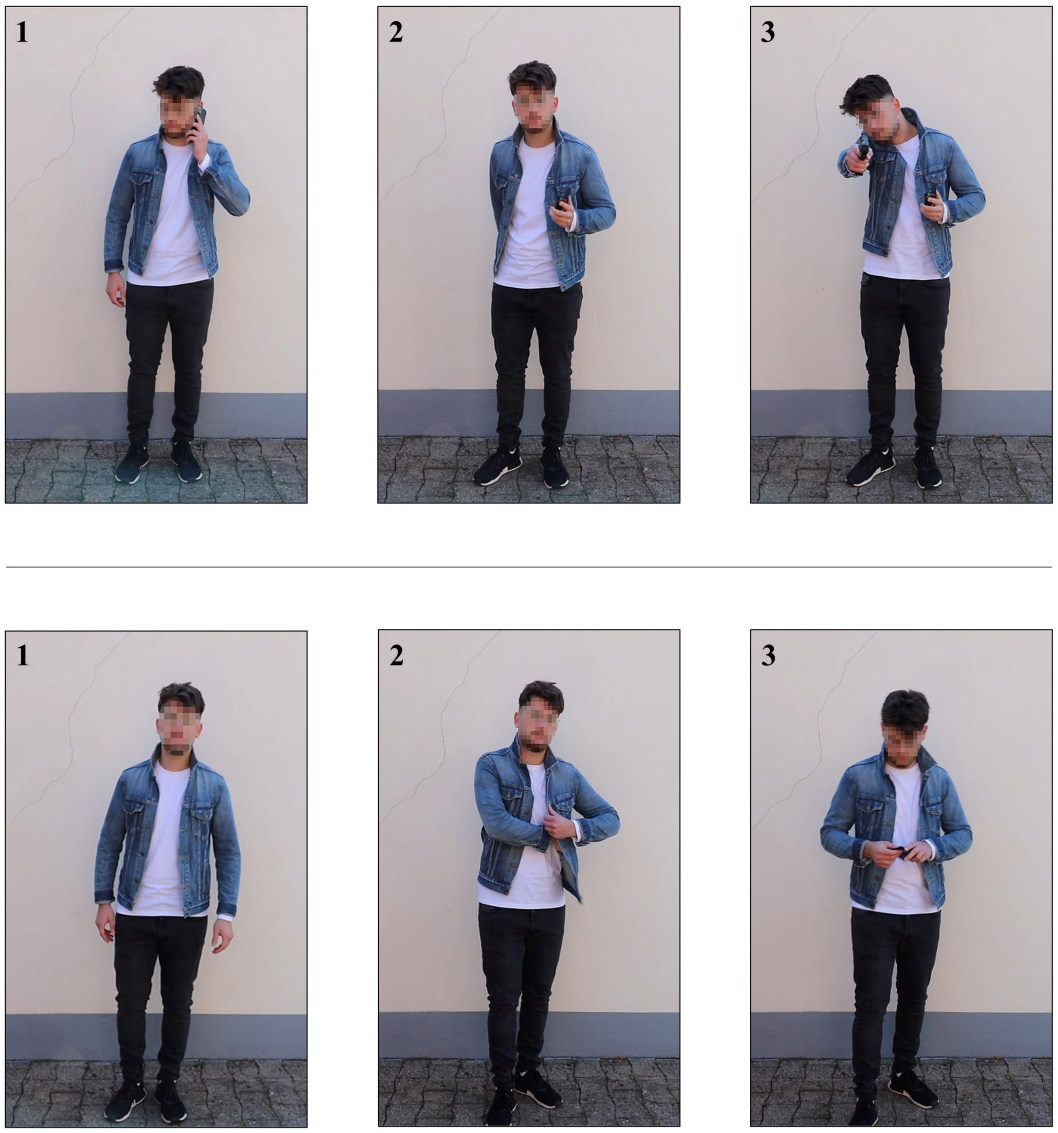
An exemplary shoot **(Upper)** and don't shoot scenario **(Lower)**. (1) Initial situation, (2) drawing motion begins, and (3) final position.

Both pre- and post-test consisted of the same two tasks as in Olma et al. (2024). Task 1 tested traditional marksmanship skills and served as a baseline and manipulation check insofar as the effect of both trainings on traditional marksmanship was to be evaluated. In task 1, the participants were instructed to first fire at two larger red circles (ø ~ 14 inches) and then at two smaller blue circles (ø ~ 7 inches), each once and as quickly and accurately as possible. They were free to choose their first red circle. Shots were not repeated in case of a miss. Task 2 tested the participants' shoot/don't shoot performance in dynamic video scenarios (Figure 1). Sixteen video scenarios with four different suspects had been developed explicitly for the previous study (for more detailed information, see Olma et al., 2024). Eight video scenarios required shoot decisions, and the other eight scenarios required don't shoot decisions. The suspects were either a young or middle-aged woman, or a young or middle-aged man. All suspects wore the same outfit: Dark shoes, dark denim jeans, a white shirt, and a blue denim jacket. In don't shoot scenarios, the suspect drew a non-hazardous object (i.e., wallet or smartphone) and presented it to the spectator. In shoot scenarios, the suspect drew a black handgun and pointed it toward the spectator. All video scenarios were roughly the same time (mean duration = 19 s) and depicted comparable motion sequences. Due to the contrast between the wall and the suspects' shirts, the black handgun could clearly be distinguished from the smartphone and the wallet. In all shoot and don't shoot scenarios, the outcome remained open until the end, but the decision to open or withhold fire was unambiguous and left no room for hesitation. The black handgun was clearly identifiable from 2 s before the end of the video scenario, because based on other studies that also included consultation with active police trainers and firearms experts (Hamilton et al., 2019; Heusler and Sutter, 2022b), this was sufficient time for both the officer and opponent to engage in lethal fire. In the pre-test, the participants were shown three different video scenarios. Only the first two were evaluated; one video scenario required a shoot decision, the other video scenario a don't shoot decision. This sequence was randomized. The third video scenario served solely as a dummy scenario that randomly required a shoot or a don't shoot decision; its purpose was to prevent participants' anticipation of how many shoot and don't shoot scenarios they would encounter. In the post-test, the participants repeated the sequence described above, but were shown three new scenarios. All video scenarios were based on the following initial situation: After an armed robbery, the participants encountered a person who fit the description of the suspect. The suspect was said to be previously convicted, possibly armed, and unpredictable. A current mugshot was shown. The participants were instructed to tactically work through the scenario. This included realistic movements, communication, shooting stance, and the use of the handgun in case of a suspect's attack, and withholding the use of the handgun in case of an unarmed suspect.

### 2.3 Training

**Figure 2:**
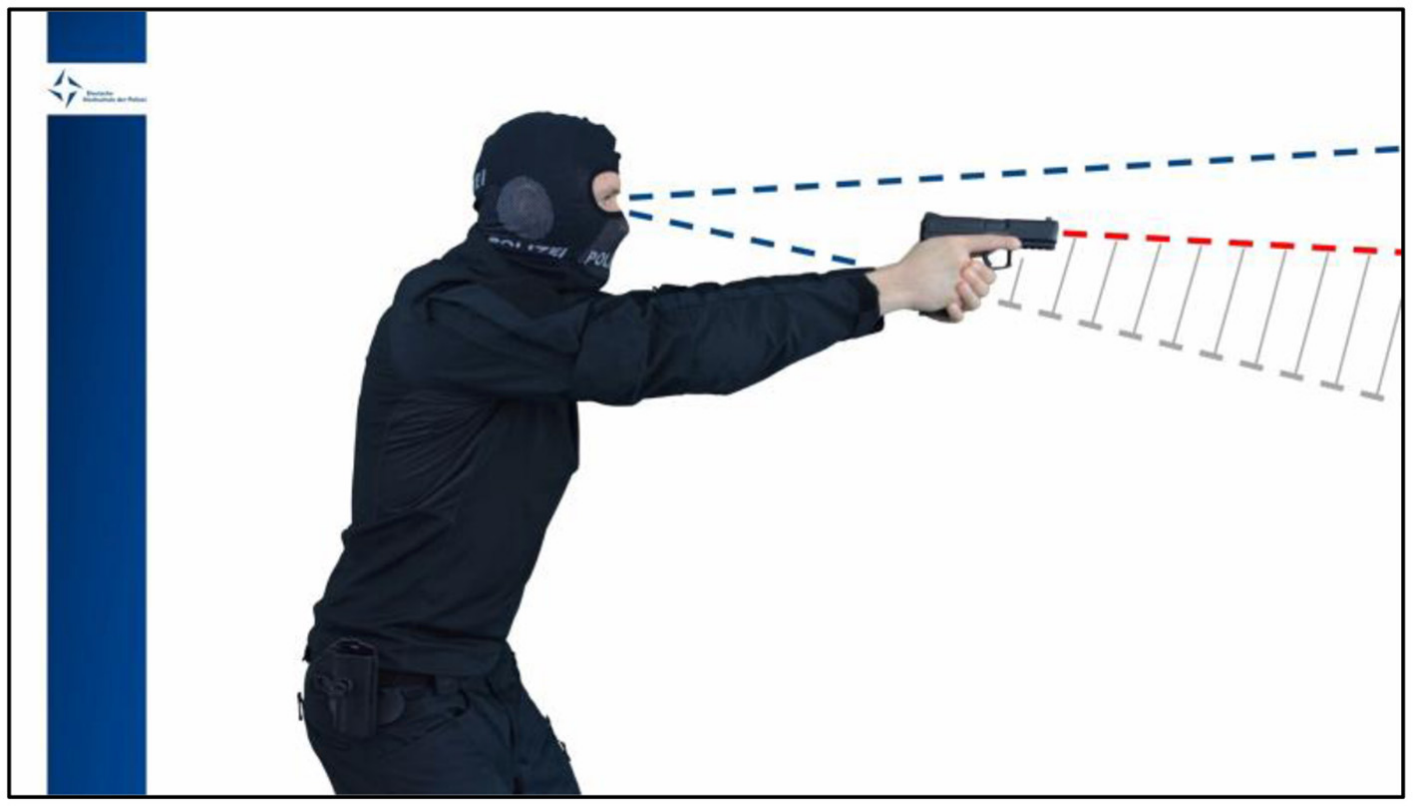
Excerpt of the educational video for the intervention training.

The same training protocol as in the previous study (Olma et al., 2024) was used: While the control training was similar to traditional police firearms training, the focus of the intervention training was on situational awareness, tactical gaze control, and visual attention. In contrast to the control training, the intervention training was embedded in a modern didactic framework based on the 4C/ID (van Merrienboer, 2020), whose purpose was to optimize the learning effect and knowledge gain despite the short time available. Table 1 describes the overall focus, learning objectives, theoretical and practical training, and the lessons learned of both training concepts. The theoretical content was delivered as a self-produced educational video—one for each group (control group = 12 min; intervention group = 18 min). At a separate desk, the seated participants were given headphones (Logitech H340) and started the respective educational video on a 15.31-inch laptop (Alienware 15 R3). Both videos followed the same structure: A hooded police firearms trainer in tactical gear welcomed the spectator and informed her/him about the focus of the training and the learning objectives. Next was the specific content, followed by a summary of the lessons learned. The control group was instructed on the speed-accuracy trade-off and optimized weapon handling, whereas the intervention group was educated on what to expect and what to be aware of when entering certain police operational situations (situational awareness), where to look (tactical gaze control), and how to distinguish hazardous from non-hazardous objects (visual attention). Table 1 describes the contents of both educational videos in further detail (for information on the creation process, see Olma et al., 2024). Figure 2 depicts an exemplary excerpt of the intervention training's educational video, in which the instructor demonstrates how hands and handgun limit one's field of view.

**Table 1:** Description of both training concepts.

	**Control training**	**Intervention training**
Focus	• Speed-accuracy trade-off	• Situational awareness • Tactical gaze control • Visual attention
Learning objectives	• Increase speed while keeping accuracy high • Optimize weapon handling • Optimize movements	• Increase competence in shoot/don't shoot decision-making • Optimize weapon stance • Optimize verbal communication
Theoretical training	• Process of shooting • Speed-accuracy trade-off • Different shooting modes • Optimal use of sights • Standard shooting errors • Optimal movement of the handgun	• Basics of visual perception • Salient stimuli • Difference between visual perception and visual attention • Situational awareness • Tactical gaze control • Speed-accuracy trade-off • Inhibitory control • Decision-making • Verbal communication
Practical training	• Exercise 1: Five rounds on square upon acoustic signal; two runs with different-sized squares • Exercise 2: One round on each of four colored squares clockwise and a finishing round in a center circle upon acoustic signal • Exercise 3: Five rounds on shrinking squares upon appearance on the canvas • Exercise 4: Five rounds on same targets as in exercise 2; order of targets was now indicated by their color; two runs with different orders • Exercise 5: Five rounds on a bullseye target upon acoustic signal	• Exercise 1: Six rounds on torso-shaped rectangle upon acoustic signal • Exercise 2: Six rounds on circles in the order of visual impulse; two runs with different orders • Exercise 3: Two rounds on human silhouette upon detection of handgun pictogram in its hand; 14 runs with different object pictograms, order, and location of the silhouette on the canvas each • Exercise 4: Two rounds on life-sized images of real persons upon detection of handgun; four runs with different images and order of appearance each
Lessons learned	• Speed and accuracy are interrelated • Find “sweet spot” for trade-off • Focus on weapon's sight • Execute movement sequences neatly • Reduce time for movement sequences	• Visual perception ≠ visual attention • Focus on hands and hip region • Keep field of vision clear, muzzle down, and both eyes open • Internalize situational awareness • Use verbal communication

For the practical exercises, the participants used the same handgun as in the pre- and post-test. All participants were given direct feedback and step-by-step instruction on their shooting performance (i.e., decisions, technique, etc.). The participants were encouraged to apply the acquired information from the educational video in the exercises. The control group had to shoot predefined targets that differed in shape, location, and size but always remained abstract stimuli, whereas the intervention group worked on exercises that gradually shifted from abstract stimuli to realistic images of suspects. A precise description of each group's practical training can be found in Table 1.

### 2.4 Procedure

In preparation for the participants, the equipment was disinfected, the magazines were reloaded with bullets and gas, the bullet holes were taped with patches, the paper canvas was unrolled, and new written materials were laid out. The respective experimental presentation was started on the paper canvas and the laptop was prepared with the respective educational video. At the beginning, all participants were introduced to the subject of the experiment and then asked for their written consent and demographic data. Only after the post-test, they were informed about random group assignment. Participants wore a service belt and a holster; they were free to take off their tactical vests. In preparation for the pre-test, the participants were given a safety briefing and time to familiarize themselves with the handgun and its specifics. When ready, two full magazines (one for the magazine pouch, each 15 rounds) were handed to the participants, and they were equipped with the mobile eye-tracking device, which was stored in either a chest or leg pocket. It was made sure that the connecting cable between the glasses and the smartphone did not impede arm movement. Prior to the first task, the participants were asked to take position behind the marking, the light was switched off, and all recordings were started. Only then participants were allowed to load the handgun. With a remote presenter, the experimental presentation was controlled. The two tasks of the pre-test were preceded by a written, detailed instruction, while a 3-s countdown preceded the target presentation. Additionally, the tasks were explained verbally if necessary. The pre-test lasted 5 min. After the pre-test, the recordings were stopped, the light was switched on, and the magazines, the handgun, and the eye-tracking device were returned. The participants then sat at the table with the laptop and received the theoretical video training. In the meantime, the magazines were reloaded, the bullet holes were taped off, and the respective training presentation was started. For practical training, participants wore safety glasses (instead of the mobile eye-tracking device) and were provided with a full magazine for each exercise. The lights were switched off again. Combined, the theoretical and practical training lasted around 35–40 min. The post-test followed the same procedure as the pre-test, and lasted another 5 min. At the end of the experiment, the participants handed back all the equipment, were informed about its objective, and had the opportunity to give feedback. Then, preparation for the next participant was initiated. In total, the experiment lasted 50 min per participant. Data were collected in June and July 2024.

### 2.5 Dependent variables

The dependent variables were the same as in the previous study (Olma et al., 2024). In task 1, the *Hit Factor* was measured as a manipulation check and to test hypothesis 6. The *Hit Factor* serves as a common indicator of marksmanship and is calculated by dividing the number of hits (max. one hit per circle, max. four hits in total) by the time taken (in seconds). For instance, three hits in exactly 4 s result in a *Hit Factor* equal to 0.75. The quotient can exceed 1.00. The higher the value, the better the performance.

In task 2, *Decisions, Response Time, First Hit, Muzzle Position*, and *Closed Eye(s)* were measured. The variable *Decisions* served to test hypothesis 3 by reflecting the participants' decision-making progress from pre- to post-test for the shoot and don't shoot scenarios separately. In the shoot scenarios, the participants either fired upon detection of the handgun, which was considered correct positive, or did refrain from shooting (i.e., no shot even though the black handgun was clearly visible for 2 s or a shot before the black handgun was clearly identifiable), which was considered false negative. The same logic applied to the don't shoot scenarios; holding back the fire was considered correct negative, and firing at the unarmed suspect false positive. Each false decision was scored with “0” and each correct one with “1”. By subtracting the pre-test score from the post-test score, a value that indicated each participant's progress (“−1” = deteriorated, “0” = constant, “+1” = improved) was calculated. The variable *Response Time* served to test hypothesis 4 by reflecting the time (in milliseconds) between the detection of the black handgun and the participants' initial motor response (shot taken on the paper canvas). Effective hits on the suspect were irrelevant to this measure. For the analysis, the moment when the black handgun was first clearly identifiable was time-stamped and the time until the first shot penetrated the paper canvas was calculated. If the participants did not shoot, their results were disregarded. The variable *First Hit* served to test hypothesis 5 by reflecting the time (in milliseconds) between the detection of the black handgun and the first effective hit in predefined areas (the suspects' torso or head). The variable's calculation is analogous to *Response Time*. If the participants did not hit the predefined areas, their results were disregarded. The variable *Muzzle Position* served to test hypothesis 1 by reflecting the time (in milliseconds) that the participants held the handgun at eyesight level and pointed it at the suspect before taking the first shot. A tactical high ready position was not considered eyesight level. Did participants shoot before bringing their handgun at eyesight level, their results were disregarded. The variable *Closed Eye(s)* served to test hypothesis 2 by reflecting the time (in milliseconds) that the participants had at least one eye closed before taking the first shot. Did participants keep both eyes open, their results were disregarded.

### 2.6 Design and statistical analyses

The study was conducted under the Declaration of Helsinki and the Ethical Principles and Protocol Code of the German Association of Psychologists [adapted from the American Psychological Association (APA) Code of Ethics]. The Ethics Committee of the German Police University (“*Ethik-Kommission der Deutschen Hochschule der Polizei”*) provided approval of the study (approval number: “DHPol-EthK.2024.Olm4”). The study was preregistered (https://aspredicted.org/95X_B51).

Analogous to the previous study (Olma et al., 2024), the experiment tested in a randomized controlled trial; 2 (group) × 2 (measurement time). The intervention group received the specified firearms training, while the control group was given an active control training in line with the common German police standard (see Section 2.3). Again, both training concepts included a theoretical and a practical part. In the pre- and post-test, each group's shooting performance was measured (see Section 2.2). The dependent variables are described in Section 2.5.

The statistical analyses were carried out using the software RStudio and with α = 5% as the significance level. For all dependent variables except *Decisions*, a repeated measures ANOVA with the within-subject factor *Time* and the between-subject factor *Group* was calculated for each dependent variable separately. When reasonable, a separate repeated measures ANOVA with the factor *Time* for each group was calculated to determine its individual progress. For *Decisions*, a non-parametric Wilcoxon Rank Sum Test was chosen.

The number of excluded data sets per dependent variable was in line with Olma et al. (2024): For the *Hit Factor* analysis, 1 data set had to be excluded due to a technical failure (canvas camera). *N* = 51 evaluable data sets remained (control group = 25; intervention group = 26). For the *Response Time* analysis, the data sets of six participants who did not shoot in at least one of the shoot scenarios (false negatives) and of another two participants who shot too early (i.e., before the handgun was clearly visible) in at least one of the shoot scenarios had to be excluded. *N* = 44 evaluable data sets remained (control group = 19; intervention group = 25). For the *First Hit* analysis, the data sets of the same six false negatives, the same two participants who shot too early, and another 10 participants who did not hit the predefined areas in at least one of the shoot scenarios had to be excluded. *N* = 34 evaluable data sets remained (control group = 15; intervention group = 19). For the *Muzzle Position* analysis, the data sets of the six false negatives, the two participants who shot too early, another three participants for which data was unavailable due to a technical failure (eye-tracking device), and all eight participants who shot before bringing the handgun up at eyesight level in at least one of the shoot scenarios had to be excluded. *N* = 33 evaluable data sets remained (control group = 15; intervention group = 18). For the *Closed Eye(s)* analysis, the data sets of the six false negatives, the two participants who shot too early, and another 35 participants who kept both eyes open before taking the shot in the pre- and post-test had to be excluded. *N* = 9 evaluable data sets remained (control group = 3; intervention group = 6).

Exploratory analyses were run to provide a more detailed insight into the effect of the intervention training on different samples. Therefore, the aforementioned statistical analyses were repeated, including only the intervention group of the present study and the intervention group of the previous study (Olma et al., 2024). For all dependent variables except for *Decisions*, a repeated measures ANOVA with the within-subject factor *Time* (pre-test vs. post-test) and the between-subject factor *Sample* (cadets vs. senior officers) was calculated for each dependent variable separately. For *Decisions*, a non-parametric Wilcoxon Rank Sum Test was chosen. In line with Olma et al. (2024), further exploratory analyses were conducted on the stimulus material, the shooting characteristics and the effect of the number of *Lessons* and the self-estimated handgun *Proficiency* on the sample's performance. Those results are reported in Supplementary material but referred to in the discussion (see Section 4.1).

## 3 Results

### 3.1 Manipulation check

For *Hit Factor*, the repeated measures ANOVA (*Time* × *Group*) showed a significant, small effect for the factor *Time* [*F*_(1, 98)_ = 5.23, *p* = 0.024; $\eta_{\text{p}}^{2}$ = 0.05, 90% CI = (0.01, 0.13)] and a non-significant, small effect for the factor *Group* [*F*_(1, 98)_ = 0.44, *p* = 0.509; $\eta_{\text{p}}^{2}$ < 0.01, 90% CI = (0.00, 0.05)]. An interaction of the factors *Time* × *Group* could not be shown [*F*_(1, 98)_ = 0.39, *p* = 0.536; $\eta_{\text{p}}^{2}$ < 0.01, 90% CI = (0.00, 0.05)]. The overall performance improved from pre- to post-test (*M*_pre_ = 0.63, *SD*_pre_ = 0.21; *M*_post_ = 0.72, *SD*_post_ = 0.19; *min*_pre_ = 0.17, *max*_pre_ = 1.00; *min*_post_ = 0.28, *max*_post_ = 1.14).

### 3.2 Main results

For *Decisions*, both groups' performance in shoot scenarios (mean progress of control group = 0.16, *SD* = 0.47; mean progress of intervention group = 0.07, *SD* = 0.27; *W* = 367.50, *p* = 0.388) and don't shoot scenarios (mean progress of control group = 0; mean progress of intervention group = 0) did not differ significantly between pre- to post-test. Table 2 shows the ratio of correct positive and false negative decisions (shoot scenarios) and correct negative and false positive decisions (don't shoot scenarios) for the control group (*n* = 25) and the intervention group (*n* = 27) per measurement time.

**Table 2:** Ratio of correct and false decisions for both groups.

	**Pre-test**		**Post-test**	
	**Control group**	**Intervention group**	**Control group**	**Intervention group**
Correct positive	20	25	24	27
False negative	5	2	1	0
Correct negative	25	27	25	27
False positive	0	0	0	0

**Figure 3:**
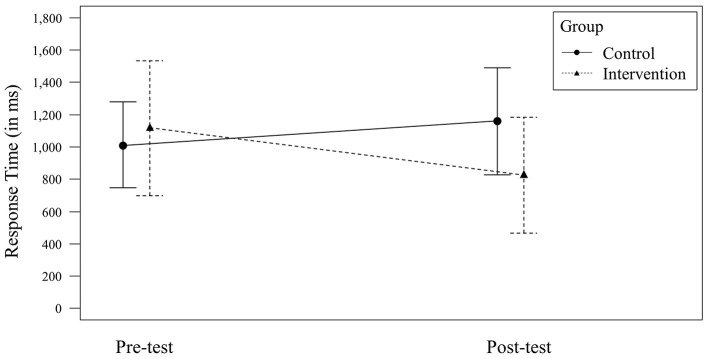
Significant *Time* × *Group* interaction effect for *Response Time*. Error bars represent the 95% CI for the mean.

For *Response Time*, a non-significant, small effect for the factor *Time* [*F*_(1, 84)_ = 1.81, *p* = 0.183; $\eta_{\text{p}}^{2}$ = 0.02, 90% CI = (0.00, 0.09)] and a non-significant, small effect for the factor *Group* [*F*_(1, 84)_ = 2.30, *p* = 0.133; $\eta_{\text{p}}^{2}$ = 0.03, 90% CI = (0.00, 0.10)] were observed. The interaction of the factors *Time* × *Group* was significant with a medium effect size [*F*_(1, 84)_ = 8.19, *p* = 0.005; $\eta_{\text{p}}^{2}$ = 0.09, 90% CI = (0.02, 0.19); see Figure 3]. On a group level, the control group (*n* = 19) did not significantly improve its *Response Time* [*F*_(1, 36)_ = 2.26, *p* = 0.141; $\eta_{\text{p}}^{2}$ = 0.06, 90% CI = (0.00, 0.21); *M*_pre_ = 1,012 ms, *SD*_pre_ = 265; *M*_post_ = 1,159 ms, *SD*_post_ = 332], whereas the intervention group (*n* = 25) did [*F*_(1, 48)_ = 6.91, *p* = 0.012; $\eta_{\text{p}}^{2}$ = 0.13, 90% CI = (0.02, 0.27); *M*_pre_ = 1,115 ms, *SD*_pre_ = 418; *M*_post_ = 824 ms, *SD*_post_ = 361].

**Figure 4:**
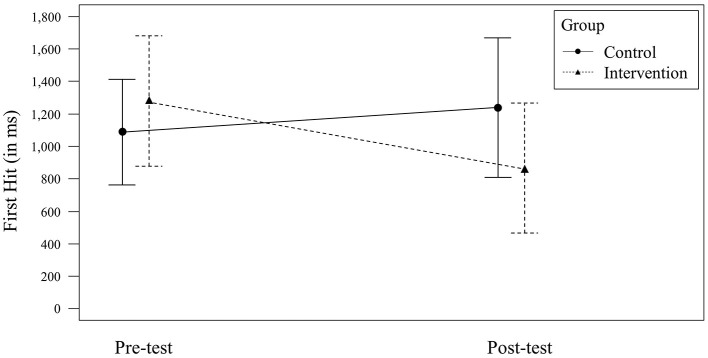
Significant *Time* × *Group* interaction effect for *First Hit*. Error bars represent the 95% CI for the mean.

For *First Hit*, a non-significant, small effect for the factor *Time* [F_(1, 64)_ = 2.97, *p* = 0.090; $\eta_{\text{p}}^{2}$ = 0.04, 90% CI = (0.00, 0.15)] and a non-significant, small effect for the factor *Group* [*F*_(1, 64)_ = 0.88, *p* = 0.351; $\eta_{\text{p}}^{2}$ = 0.01, 90% CI = (0.00, 0.09)] were observed. The interaction of the factors *Time* × *Group* was significant with a medium effect size [*F*_(1, 64)_ = 8.60, *p* = 0.005; $\eta_{\text{p}}^{2}$ = 0.12, 90% CI = (0.02, 0.24); see Figure 4]. On a group level, the control group (*n* = 15) did not significantly improve its *First Hit* [*F*_(1, 28)_ = 1.17, *p* = 0.289; $\eta_{\text{p}}^{2}$ = 0.04, 90% CI = (0.00, 0.19); *M*_pre_ = 1,088 ms, *SD*_pre_ = 326; *M*_post_ = 1,238 ms, *SD*_post_ = 431], whereas the intervention group (*n* = 19) did [*F*_(1, 36)_ = 10.01, *p* = 0.003; $\eta_{\text{p}}^{2}$ = 0.22, 90% CI = (0.05, 0.39); *M*_pre_ = 1,279 ms, *SD*_pre_ = 403; *M*_post_ = 866 ms, *SD*_post_ = 402].

For *Muzzle Position*, a non-significant, small effect for the factor *Time* [*F*_(1, 62)_ = 1.81, *p* = 0.183; $\eta_{\text{p}}^{2}$ = 0.03, 90% CI = (0.00, 0.12)] and a non-significant, small effect for the factor *Group* [*F*_(1, 62)_ = 0.97, *p* = 0.330; $\eta_{\text{p}}^{2}$ = 0.02, 90% CI = (0.00, 0.10)] were observed. The interaction of the factors *Time* × *Group* lacked significance with a small effect size [*F*_(1, 62)_ = 2.67, *p* = 0.107; $\eta_{\text{p}}^{2}$ = 0.04, 90% CI = (0.00, 0.15)]. Table 3 shows that the control group (*n* = 15) prolonged the time of having the handgun at eyesight level before shooting, whereas the intervention group (*n* = 18) reduced that time.

**Table 3:** Mean *Muzzle Position* (in ms) for both groups for pre- and post-test.

	**Pre-test**	**Post-test**
Control group	160 (*SD* = 204)	205 (*SD* = 176)
Intervention group	424 (*SD* = 723)	139 (*SD* = 147)

For *Closed Eye(s)*, a non-significant, medium effect for the factor *Time* [*F*_(1, 14)_ = 1.23, *p* = 0.287; $\eta_{\text{p}}^{2}$ = 0.08, 90% CI = (0.00, 0.32)] and a non-significant, negligible effect for the factor *Group* [*F*_(1, 14)_ = 0.00, *p* = 0.999; $\eta_{\text{p}}^{2}$ < 0.01, 90% CI = (0.00, 0.00)] were observed. The interaction of the factors *Time* × *Group* lacked significance with a small effect size [*F*_(1, 14)_ = 0.90, *p* = 0.358; $\eta_{\text{p}}^{2}$ = 0.06, 90% CI = (0.00, 0.30)]. Table 4 shows that both the control group (*n* = 3) and the intervention group (*n* = 6) prolonged the time of having at least one eye closed before shooting.

**Table 4:** Mean *Closed Eye(s)* (in ms) for both groups for pre- and post-test.

	**Pre-test**	**Post-test**
Control group	78 (*SD* = 107)	267 (*SD =* 240)
Intervention group	155 (*SD* = 78)	189 (*SD* = 203)

### 3.3 Exploratory analyses: comparison of police cadets and senior police officers

For *Hit Factor*, the repeated measures ANOVA (*Time* × *Sample*) showed a significant, medium effect for the factor *Time* [*F*_(1, 92)_ = 8.57, *p* = 0.004; $\eta_{\text{p}}^{2}$ = 0.09, 90% CI = (0.02, 0.18)] and a non-significant, small effect for the factor *Sample* [*F*_(1, 92)_ = 1.35, *p* = 0.249; $\eta_{\text{p}}^{2}$ = 0.01, 90% CI = (0.00 0.08)]. An interaction of the factors *Time* × *Sample* could not be shown [*F*_(1, 92)_ = 0.64, *p* = 0.426; $\eta_{\text{p}}^{2}$ = 0.01, 90% CI = (0.00, 0.06)]. Table 5 shows the improvement of the cadets (*n* = 26) and the senior officers (*n* = 22) from pre- to post-test.

**Table 5:** Mean *Hit Factor* for both samples for pre- and post-test.

	**Pre-test**	**Post-test**
Cadets	0.60 (*SD* = 0.25)	0.72 (*SD* = 0.19)
Senior officers	0.62 (*SD* = 0.34)	0.82 (*SD* = 0.26)

For *Decisions*, both groups' performance in shoot scenarios (mean progress of cadets = 0.07, *SD* = 0.27; mean progress of senior officers = 0.18, *SD* = 0.39; *W* = 329.00, *p* = 0.265) and don't shoot scenarios (mean progress of cadets = 0; mean progress of senior officers = 0) did not differ significantly between pre- to post-test. Table 6 shows the ratio of correct positive and false negative decisions (shoot scenarios), and correct negative and false positive decisions (don't shoot scenarios) for the cadets (*n* = 27) and the senior officers (*n* = 22) per measurement time.

**Table 6:** Ratio of correct and false decisions for both samples.

	**Pre-test**		**Post-test**	
	**Cadets**	**Senior officers**	**Cadets**	**Senior officers**
Correct positive	25	17	27	21
False negative	2	5	0	1
Correct negative	27	22	27	22
False positive	0	0	0	0

For *Response Time*, a significant, large effect for the factor *Time* [*F*_(1, 80)_ = 17.20, *p* < 0.001; $\eta_{\text{p}}^{2}$ = 0.18, 90% CI = (0.07, 0.30)] and a significant, medium effect for the factor *Sample* [*F*_(1, 80)_ = 6.22, *p* = 0.015; $\eta_{\text{p}}^{2}$ = 0.07, 90% CI = (0.01, 0.17)] were observed. An interaction of the factors *Time* × *Sample* could not be shown [*F*_(1, 80)_ = 0.57, *p* = 0.452; $\eta_{\text{p}}^{2}$ = 0.01, 90% CI = (0.00, 0.07)]. Table 7 shows the improvement of the cadets (*n* = 25) and the senior officers (*n* = 17) from pre- to post-test.

**Table 7:** Mean *Response Time* (in ms) for both samples for pre- and post-test.

	**Pre-test**	**Post-test**
Cadets	1,115 (*SD* = 418)	824 (*SD* = 361)
Senior officers	969 (*SD* = 421)	551 (*SD* = 280)

For *First Hit*, a significant, large effect for the factor *Time* [*F*_(1, 58)_ = 16.01, *p* < 0.001; $\eta_{\text{p}}^{2}$ = 0.22, 90% CI = (0.08, 0.35)] and a non-significant, small effect for the factor *Sample* [*F*_(1, 58)_ = 2.35, *p* = 0.131; $\eta_{\text{p}}^{2}$ = 0.04, 90% CI = (0.00, 0.14)] were observed. An interaction of the factors *Time* × *Sample* could not be shown [*F*_(1, 58)_ = 0.11, *p* = 0.740; $\eta_{\text{p}}^{2}$ < 0.01, 90% CI = (0.00, 0.05)]. Table 8 shows the improvement of the cadets (*n* = 19) and the senior officers (*n* = 12) from pre- to post-test.

**Table 8:** Mean *First Hit* (in ms) for both samples for pre- and post-test.

	**Pre-test**	**Post-test**
Cadets	1,279 (*SD* = 403)	866 (*SD* = 402)
Senior officers	1,143 (*SD* = 555)	654 (*SD* = 401)

For *Muzzle Position*, a non-significant, small effect for the factor *Time* [*F*_(1, 56)_ = 1.67, *p* = 0.202; $\eta_{\text{p}}^{2}$ = 0.03, 90% CI = (0.00, 0.13)] and a significant, medium effect for the factor *Sample* [*F*_(1, 56)_ = 4.79, *p* = 0.033; $\eta_{\text{p}}^{2}$ = 0.08, 90% CI = (0.01, 0.20)] were observed. An interaction of the factors *Time* × *Sample* could not be shown [*F*_(1, 56)_ = 0.66, *p* = 0.422; $\eta_{\text{p}}^{2}$ = 0.01, 90% CI = (0.00, 0.09)]. Table 9 shows the improvement of the cadets (*n* = 18) and the senior officers (*n* = 12) from pre- to post-test.

**Table 9:** Mean *Muzzle Position* (in ms) for both samples for pre- and post-test.

	**Pre-test**	**Post-test**
Cadets	424 (*SD* = 723)	139 (*SD* = 147)
Senior officers	1,806 (*SD* = 3,679)	774 (*SD* = 1,104)

## 4 Discussion

### 4.1 Discussion of main results

The present study aimed to replicate the previous study (Olma et al., 2024) with a sample of second-year police cadets to validate the efficacy of the individual video-based intervention training across different levels of experience and expertise. In this preregistered lab experiment, the active control group received a traditional police firearms training, whereas the intervention group's training focused on situational awareness, tactical gaze control, and visual attention. In the pre- and post-test, the participants had to demonstrate their traditional marksmanship skills and performance in dynamic shoot/don't shoot video scenarios. Overall, the findings from the previous study were replicated successfully; again, both groups performed close to the performance maximum, but beyond that, the intervention group was able to significantly improve its response execution. Moreover, an increased sample size enhanced the outcome's validity.

Against hypothesis 6, the improvement of the *Hit Factor* from pre- to post-test can be explained exclusively by *Time*, not by *Group*. Although the *Hit Factor* was intended to serve as a manipulation check, the control group, after receiving the training focused on the speed-accuracy trade-off, performed comparably well, but not better, when compared to the intervention group. This result contradicts the findings of Heusler and Sutter (2022b). In the previous study (Olma et al., 2024), it was assumed that experience and expertise leveled out the added value of the traditional firearms training delivered to the control group. Since this explanation is not applicable to the present study, it seems more likely that in the intervention group, learning effects from the pre-test and/or training effects from the intervention (with complex and dynamic decision scenarios) transferred to the basic hit task—an unintended side-effect that is also reported in sports (Deuker et al., 2024). Future studies are necessary to disentangle pre-post learning effects from intervention-post transfer effects.

Furthermore, it was hypothesized that the intervention group would improve their *Decisions* in the shoot and don't shoot scenarios to a greater extent than the control group (hypothesis 3). Yet again, this hypothesis had to be rejected because the error rate was marginal: For the shoot scenarios, the level of correct decisions was initially high, with a slight advantage in favor of the intervention group. In the post-test, decision-making was very accurate with only one participant in the control group who refrained from shooting (false negative). For the don't shoot scenarios, decision-making was maximally accurate, and false positives cannot be reported for any measurement time. Hence, no significant differences in decision-making between the two groups were observed. The initially low number of false negative and no false positive decisions undercuts other studies by far (Biggs et al., 2015; Hamilton et al., 2019; Heusler and Sutter, 2022b; Vickers and Lewinski, 2012). While this finding was also attributed to experience and expertise in the previous study, the explanation for the present sample of cadets must be more complex: On the one hand, the decision to shoot/not to shoot might have such serious and drastic consequences that participants chose a conservative approach. Targeted training, individual inhibitory control, and the level of situational awareness and anticipation greatly impact the quality of decision-making: At the expense of few false negatives, the participants eliminated false positive decisions that would have resulted in harming innocent suspects. On the other hand, the results confirm the impression that the participants engaged and tactically worked through the scenarios. Yet, generalizing the outcome for *Decisions* urges caution: Despite considerable levels of dynamism and uncertainty, the participants were not exposed to stress and pressure usually caused by live fire training, force-on-force setups or increased cognitive load (Biggs and Pettijohn, 2021; Heusler and Sutter, 2022b; Kleider-Offutt et al., 2016; Oudejans, 2008; Vickers and Lewinski, 2012).

In line with hypothesis 4, the significant interaction for *Response Time* confirms that after training, the intervention group shot faster in shoot scenarios than the control group. The intervention group significantly improved their performance from pre- to post-test while the control group's performance remained stable. Apparently, the intervention training enabled participants to react faster after they had made the correct decision, presumably due to enhanced gaze patterns (e.g., focusing on a suspect's hands and hip region). This finding is in line with previous studies (cf. Heusler and Sutter, 2022b; Nieuwenhuys and Oudejans, 2011).

Consequently, the significant interaction for *First Hit* confirms hypothesis 5: After training, the intervention group did not only shoot faster but also took less time to hit an armed suspect in the predefined areas than the control group. It comes as little surprise that participants who shot faster also hit faster; this outcome provides further evidence that situational awareness, tactical gaze control, and visual attention not only have a positive effect on response speed but also its accuracy (speed-accuracy trade-off).

Moreover, it was hypothesized that the intervention group would bring their handgun to eyesight level later than the control group (*Muzzle Position*, hypothesis 1). Although descriptively, the intervention group decreased that time while the control group prolonged, these marginal differences proved statistically non-significant. Both groups had already kept their field of vision as free as possible in the pre-test. This tactical conduct might already be taught in regular lessons, in which they had also been sensitized not to draw the service weapon instantly or to point it at the counterpart unless inevitable (cf. Taylor, 2020).

A non-significant interaction for *Closed Eye(s)* was observed and its statistical value is further limited due to the small dataset. The possible explanation for this outcome goes hand-in-hand with the aforementioned *Muzzle Position*. Even if the complementary knowledge about the optimized processing of visual information conveyed by the educational video did not affect the *Muzzle Position* or *Closed Eye(s)*, there is reason to assume that the intervention group consciously or unconsciously utilized this knowledge to improve its response execution.

The non-significant results for the effect of *Lessons* and *Proficiency* on the dependent variables underline the homogeneity of the sample (see Supplementary material). Despite small differences in the firearms lessons taken, the performance in the pre- and post-test did not seem to depend on it. As the participants rated their proficiency with their service weapon individually, typical biases (social desirability, central tendency error, etc.) might have further reduced variance.

In addition, the effect of the stimulus material on the participants' performance was explored (see Supplementary material). In the pre-test, significant interactions of *Group* and *Stimulus Sequence* for *Response Time* and *First Hit* were observed: On the one hand, the intervention group shot and hit the suspect faster than the control group when the first video scenario was a shoot scenario, but on the other hand, took longer when the first video was a don't shoot scenario and only the second video demanded shooting. As both interaction effects dissolved in the post-test, those findings are probably mere coincidences. The significant main effect of *Stimulus Sequence* on *Muzzle Position* in the pre-test is plausible: As both groups did not exactly know what to expect and how to react properly in the first scenario, they brought their handgun to eyesight level quite early if they encountered an armed suspect (shoot scenario). Conversely, if they were displayed a don't shoot scenario first, they might have anticipated the upcoming scenario and the correct response (shooting), which they executed calmly and less hectic. In the post-test, this effect did not persist—presumably due to learning effects in both groups and the explicit contents of the training in the intervention group. Furthermore, in the pre-test, all false negative decisions across both groups were made in scenarios displaying the younger female and the older male. This effect of *Subject* on *Decisions* also dissolved in the post-test but raised questions as to the cause of this unequal pattern: According to literature (cf. Ba et al., 2021; Edwards et al., 2019; Wright and Headley, 2020), the variables age and gender indeed influence the experience of violence and being shot by police; the probability is highest for young and/or male suspects. However, the participants did not refrain from shooting not exclusively in scenarios with female or old suspects. Since all scenarios were standardized and displayed in random and counterbalanced order, this finding is less likely to be a bias but rather another coincidence. Considering the information on the shooting characteristics (see Supplementary material) draws a clear picture: There is no statistical evidence that either group outperformed the other in either measurement time. This leads to several conclusions: First, both groups' baseline level of marksmanship skills was fairly high and left limited leeway for improvement. Second, the absence of significant main or interaction effects does not prove the ineffectiveness of the control training. Third, and conversely, the assumption that the intervention training is equally beneficial to traditional marksmanship skills can neither be confirmed nor rejected.

### 4.2 Comparison of police cadets and senior police officers

In the introduction, the terms experience and expertise were outlined (Bransford and Cocking, 2000; Chi et al., 1988). Given this distinction, it is reasonable to assume that the sample of senior police officers from the previous study (Olma et al., 2024) had expertise (due to targeted training) and experience (due to years of service) with firearms, whereas the sample of police cadets from the present study had a small amount of expertise and no experience. Naturally, experience and expertise are somewhat intertwined; even if the cadets practiced the use of firearms every week (including theoretical teaching) and the senior officers demonstrated their skills less frequently, the senior officers' experience should still contribute to a superior level of expertise. Given the assumption that the present sample of cadets was inferior in terms of experience and expertise, the literature suggests that the sample of cadets should benefit from the intervention training to a greater extent than the previous sample of senior officers. Albeit, comparing the outcomes from the previous and present study did not support the aforementioned assumption: Generally, the statistical result pattern for both samples was almost identical and similar effects of the intervention training were observed. Tactical gaze control and effective visual attention were highly distinctive in both cadets and senior officers: For *Muzzle Position*, the interaction lacked significance. However, clear, descriptive differences are affirmed by a significant main effect of *Sample*, which proves that the cadets kept their eyes open longer before shooting than the senior officers in the pre- and post-test. Meaningful analyses for *Closed Eye(s)* were not possible due to the previous study's available data (*n* = 1). Based on those tactical skills, decision-making among both samples was equally accurate: Despite the descriptive superiority of the cadets, there was no evidence that either sample demonstrated greater improvement in their *Decisions* from pre- to post-test. Cadets and senior officers executed their tasks on the verge of maximum performance. The similarity of both samples is also manifested in their response execution: In absolute terms, the significant main effects of *Time* and *Sample* on *Response Time* showed that the senior officers were faster in shooting on the paper canvas than the cadets in the pre- and post-test, but in the absence of a significant interaction, a greater improvement among the senior officers cannot be ascertained. For *First Hit*, the senior officers' advantages did not persist; neither main effects nor an interaction favored either sample. However, the descriptive results indicate that a larger amount of data available might have shown another main effect of *Sample* in favor of the senior officers. For *Hit Factor*, equal scores in the pre-test led to a descriptive advantage in favor of the senior officers in the post-test. Yet, the lack of significance demands the conclusion that neither sample improved their marksmanship skills to a greater extent than the other. Upon closer examination of the data, however, it became apparent that the senior officers displayed more variance while individual scores were closer among the cadets. This most likely reflects the occupational circumstances, with the cadets having the same level of training and therefore performing on a similar level in contrast to the senior officers being appointed diverse functions within the police and therefore showing a more heterogenous performance.

The exploratory analyses of the cadets' and senior officers' performance revealed that although they demonstrated statistically different baseline levels in some variables (*Response Time* and *Muzzle Position*), none of the samples derived greater benefit from the intervention training—or at least was able to prove this in the post-test. Hence, the assumption that the intervention training must have triggered different mechanisms of action in the two samples is renewed (Olma et al., 2024): The cadets, on the one hand, had less experience and expertise which they might have compensated with up-to-datedness and intensive schooling at their police college. Latest curricula could explain the cadets' advantage in their *Muzzle Position*. Overall, the intervention training might have provided the cadets with new input and knowledge that they could use to build on and consolidate what they had learned so far. The senior officers, on the other hand, had more experience as police officers (on average 13 years) and expertise. Eventually, this might explain the superior *Response Time* (and the barely insignificant lower times until the *First Hit*). Contrary to the cadets, it is presumed that the senior officers learned little new from the intervention training but rather refreshed prior knowledge and elaborated the optimal movement sequence.

In the end, the finding that neither sample benefited more from training than the other is only disappointing from a statistical point of view. Since most participants in the present and previous study (Olma et al., 2024) were never given a blended and individual video-based teaching and had never engaged in dynamic video scenarios, the outcomes for both cadets and senior officers lead to several conclusions: First, baseline levels of performance were rather high in both samples, i.e., the regular training at the police college enabled cadets to adapt to this new environment to a similar degree as the senior officers' daily routine. Second, and in contradiction to the usual stereotype that age impacts learning success in digital education (cf. Fleming et al., 2017; Staddon, 2020), the senior officers did benefit as much from the educational approach. Third, both cadets and senior officers were able to train in non-lethal and, hence, economical environments without sacrificing considerable levels of uncertainty in police operations. Fourth, in comparison to their respective control group, the intervention training improved both samples' performance from pre- to post-test to an extent that is consistent with previous research (Heusler and Sutter, 2022b) and presumably approaches a natural performance limit that might only be surpassed by professional sport shooters (cf. Mon-López et al., 2022; Share et al., 2009). Fifth, the dichotomization of expertise was appropriate for the exploratory comparison between cadets and senior officers; for a more detailed analysis, a quantitative approach would be preferable to a qualitative one. Finally, the comparison of the two samples is afflicted with statistical uncertainties due to different environmental factors, the influence of which cannot be ruled out. Hence, the lack of significant differences between senior officers and cadets reflects a fairly probable, but not entirely definite reality; only a future study in a between-subject design could shade light on uncertainty.

### 4.3 Limitations and future studies

Overall, the replication of the results from the previous study (Olma et al., 2024) supports the assumption that the individual video-based intervention training improved performance in dynamic shoot/don't shoot scenarios. In comparison to the previous study, weapon malfunction was reduced, less experienced participants were recruited, the sample size was increased, and thus the statistical significance of the findings was enhanced. Yet, to replicate the experimental design, some of the shortcomings from the previous study had to be adopted (for further recommendations see Olma et al., 2024). In particular, the results for *Decisions* highlight two issues that were already discussed in the previous study: First, low error rates raise the question of whether the task was too easy and its cognitive demands were too low. According to the CLT and 4C/ID, a complex task should have imposed high levels of cognitive load on the participants; more variance in a non-routine skill like *Decisions* might have been observed in the pre-test since an unbalanced ratio of cognitive demands and capacity seems to deteriorate shooting performance (for a review, see Kleider-Offutt et al., 2016). Although the depiction of angry faces and handguns in the video scenarios should increase cognitive load *per se* (Bardeen and Daniel, 2017; Kret et al., 2013) and participants were instructed to verbally communicate, a more realistic and representative setting (e.g., live ammunition, threat of non-lethal counterfire, additional visual or auditive input, etc.) might have elevated stress levels, increased the task complexity, and thus revealed different outcomes. Second, despite highest efforts, the video scenarios are less dynamic and do not accurately represent a real police operation. Decision-making should be considered as an active individual-environment interaction (see Ecological Dynamics Model; Araújo et al., 2006) in which adapting to affordances and constraints occurs in nonlinear ways. Naturally, the suspects in the video scenarios would not explicitly answer or react to the participants' requests, although movement sequences and dialogues were designed to match a typical interaction. Apparently, engaging in dynamic video scenarios, that adhered to the highest scientific standards and were developed together with police practitioners, did ensure experimental standardization but reduced task fidelity. Even though this study was aimed at validating the intervention training and therefore these issues could not be addressed in the present replication, future studies are encouraged to explore further routes toward an economical yet more realistic and representative operationalization of shoot/don't shoot scenarios. This also includes the induction of stress and anxiety—factors that can significantly deteriorate performance in both training and real-world application (Eysenck et al., 2007; Landman et al., 2016; Nieuwenhuys and Oudejans, 2011).

In line with the CLT and 4C/ID, current research emphasizes the requirement for police firearms training to adapt to the needs of the cadets to optimize knowledge and skills transfer (cf. Kleygrewe et al., 2022; Staller et al., 2022, 2023). The intervention training in the present and previous study (Olma et al., 2024) provided such a blueprint: Evidence-based content was delivered by a competent police firearms trainer in a structured learning environment that prioritized the individual learner's demands (van Merrienboer, 2020). This blended learning approach also included the use of digital educational methods; in times of artificial intelligence and virtual reality, future police firearms trainings will mostly likely shift toward immersive training approaches (Caserman et al., 2022; Kleygrewe et al., 2024; Tawa et al., 2022). Although immersive training offers a variability of advantages (seemingly infinite simulation possibilities, safe environments, stress inoculation, perhaps a reduction of training costs, etc.), its use is still understudied. Future research will show whether immersive developments will be able to implement the benefits of force-on-force roleplay or live fire exercises (cf. Biggs and Pettijohn, 2021; Hamilton et al., 2019; Heusler and Sutter, 2022b).

## 5 Conclusion

All of a sudden, police officers may be confronted with life-threatening situations that demand the use of their service weapon. Optimal preparation for these unlikely events requires sound firearms training that enables correct assessment and accurate decision-making. In the present study, an individual video-based intervention training improved police cadets' performance in dynamic shoot/don't shoot scenarios more than traditional firearms control training. This blended learning approach combined theoretical input and practical implementation in a state-of-the-art didactical framework, focusing on situational awareness, tactical gaze control, and visual attention. Using a standardized educational video, carefully designed video scenarios, and a non-lethal handgun helped create an economical and low-risk setting for future police firearms training. In a previous study (Olma et al., 2024), the same two training approaches in the same experimental setup were applied to a sample of senior police officers. It was outlined that those senior officers benefited from the intervention training statistically no more or less than the cadets. Apparently, the ascribed experience and expertise did not give the senior officers a natural advantage, nor did the cadets' ascribed unbiasedness or willingness to learn favor them. Hence, the intervention training unfolded its effect through different mechanisms of action: While the cadets consolidated, the senior officers refreshed their skills and knowledge. The outcomes in both studies send a promising signal: Despite the availability of elaborate and technologically more sophisticated training facilities, the described individual video-based firearms training is a resource-efficient option to support current police training practices with supplementary content and exercises. Ultimately, the authors renew their recommendation to incorporate training approaches that devote more attention to situational awareness, tactical gaze control, and visual attention.

## Data Availability

The raw data supporting the conclusions of this article will be made available by the authors, without undue reservation.
