# Supplementary material for: Blended police firearms training improves performance in shoot/don't shoot scenarios: a systematic replication with police cadets
Source: Front Psychol. 2024 Dec 18;15:1495812. doi: 10.3389/fpsyg.2024.1495812 (PMC11689204; doi:10.3389/fpsyg.2024.1495812)
Supplement: Supplementary file 1 [file Table_1.DOCX]

Supplemental Material

# Exploratory analyses

First, we were interested in whether the four different suspects had an effect on the participants’ performance in the shoot scenarios. Hence, we calculated an ANCOVA for each measurement time with the between-subject variable *Group,* the covariate *Suspect* and the respective outcome variable. Additionally, we investigated if the sequence of the stimulus presentation (shoot – don’t shoot vs. don’t shoot – shoot) affected performance. Again, we resorted to an ANCOVA for *Stimulus Sequence* just as with *Subject*. Our participants were confronted either with a shoot or a don’t shoot scenario first (*Stimulus Sequence*) and for the shoot scenarios, we had four different suspects with two different versions each (*Suspect*). The *Stimulus Sequence* was counterbalanced across the two groups and per measurement time (pre-test: χ^2^(1, *N* = 52) < 0.01, *p* = 0.983; post-test: χ^2^(1, *N* = 52) = 0.10, *p* = 0.755) as well as the presentation of *Suspect* (pre-test: χ^2^(3, *N* = 52) = 0.40, *p* = 0.940; post-test: χ^2^(3, *N* = 52) = 1.31, *p* = 0.726; see Table 4). Neither in pre- or post-test did *Stimulus Sequence* have an effect on both groups’ *Decisions* and *Closed Eye(s).* However, in the pre-test we observed a significant interaction of the factors *Group* × *Stimulus Sequence* for *Response Time* (*F*(1,41) = 5.53, *p* = 0.024; η_p_^2^ = 0.12, 90% CI = [0.01, 0.27]) and a significant interaction of the factors *Group* × *Stimulus Sequence* for *First Hit* (*F*(1,34) = 16.19, *p* < 0.001; η_p_^2^ = 0.32, 90% CI = [0.12, 0.49]). Figure 1 demonstrates that in the pre-test, the control group responded faster when the first scenario was a don’t shoot scenario (compared to a slower response when the first scenario was a shoot scenario) whereas this effect was reversed for the intervention group. The same pattern applies to *First Hit* (Figure 2). Furthermore, *Stimulus Sequence* had a significant effect on *Muzzle Position* (*F*(1,33) = 4.36, *p* = 0.045; η_p_^2^ = 0.12, 90% CI = [0.01, 0.29]): Both groups put their gun at eyesight level significantly later when the first video scenario was a don’t shoot scenario (compared to an earlier *Muzzle Position* when the first scenario was a shoot scenario). In the post-test, both interaction effects and the main effect on *Muzzle Position* did not persist.


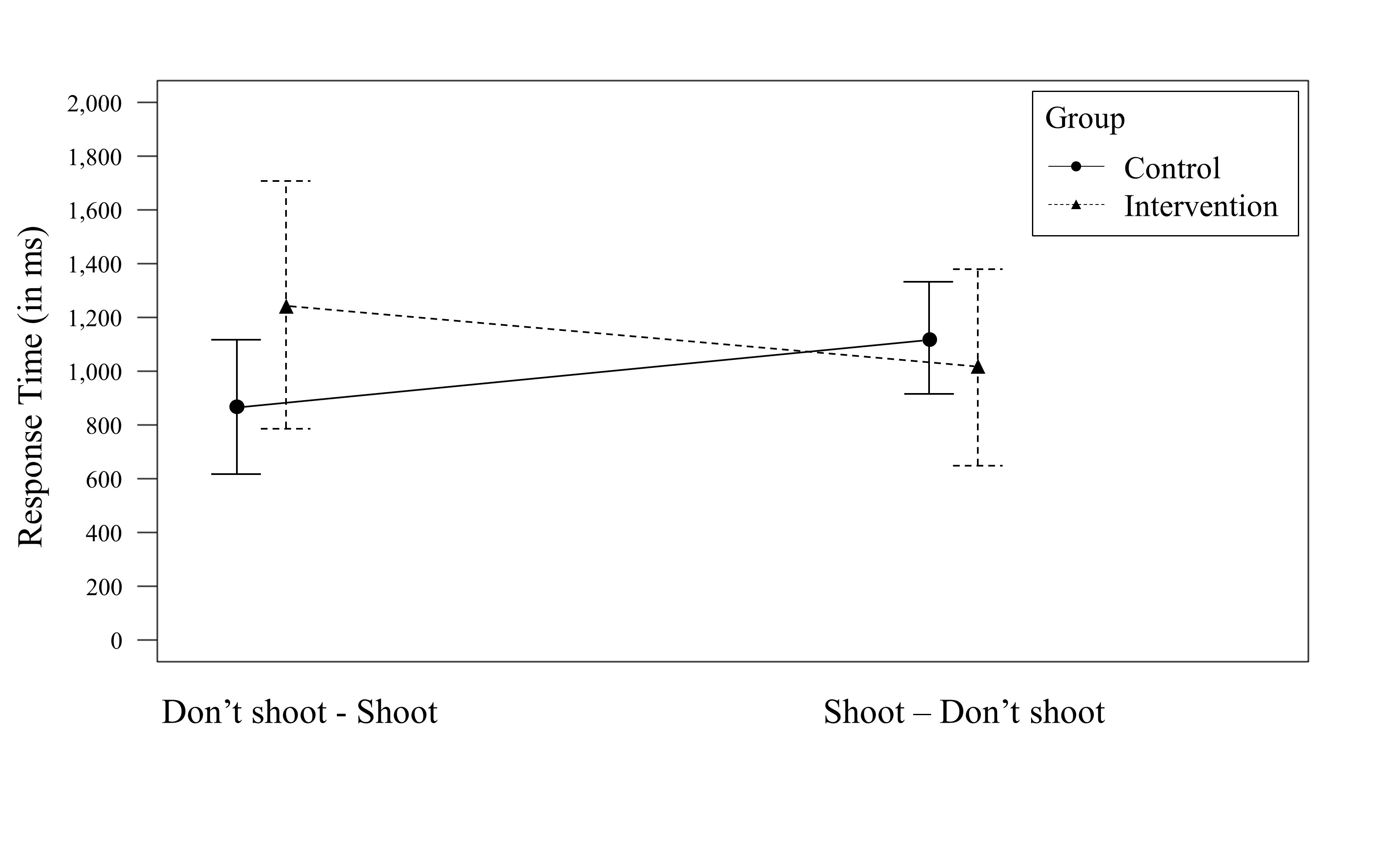


Figure 1. Significant *Group* × *Stimulus Sequence* interaction effect for *Response Time*. Error bars represent the 95% CI for the mean.


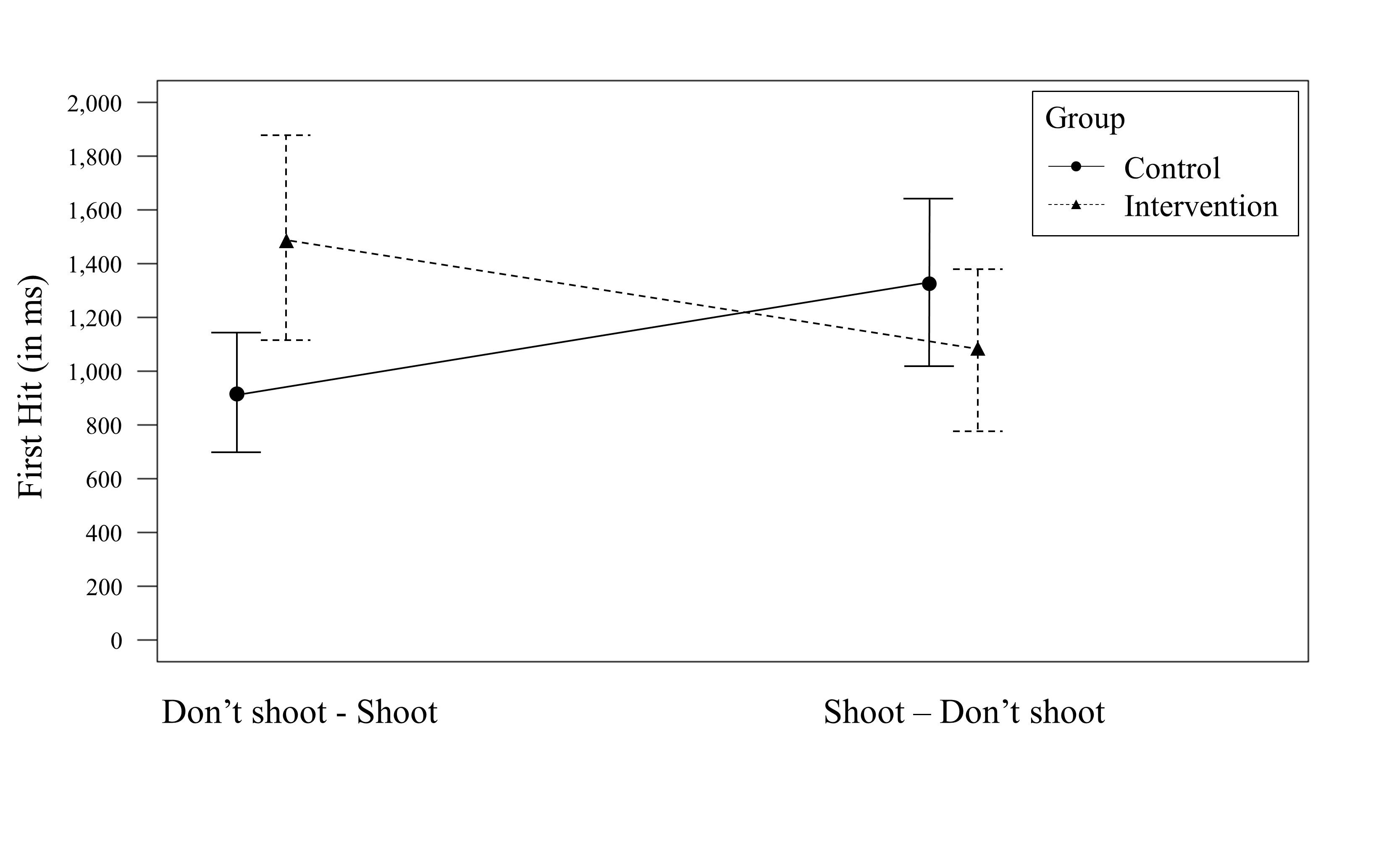


Figure 2. Significant *Group* × *Stimulus Sequence* interaction effect for *First Hit*. Error bars represent the 95% CI for the mean.

As for *Subject*, we solely observed a significant effect on the control group’s *Decisions* in the pre-test (χ^2^(3, *N* = 25) = 9.17, *p* = 0.027) where all false negative decisions occurred in scenarios with the younger female and the older male. This effect also dissolved in the post-test. Moreover, we were interested in the shooting characteristics which is why we analyzed the number of shots fired (*Shots*), the number of hits (*Hits*), its *Percentage* and which of the fired shots hit first (*Ranking*) for both groups. We calculated a repeated measures ANOVA with the within-subject factor *Time* and the between-subject factor *Group* for *Shots* and *Hits*. For Ranking, we used a two-sided Fisher-test. Both the analyses on the stimulus material and the shooting characteristics were limited to the shoot scenarios. Table 5 displays more detailed information of task 2. Per group and measurement time, the total number of *Shots* and the corresponding number of *Hits* result in a *Percentage*. *Ranking* indicates which of the participants’ shot hit the predefined area.

Table 5. Shooting characteristics progress for both groups for pre- and post-test.

|  | **Pre-test** | | **Post-test** | |
| --- | --- | --- | --- | --- |
|  | Control group  (*n* = 20) | Intervention group  (*n* = 25) | Control group  (*n* = 24) | Intervention group  (*n* = 27) |
| *Shots* | Total = 32  Mean = 1.60  (*SD* = 0.68) | Total = 58  Mean = 2.32  (*SD* = 1.46) | Total = 49  Mean = 2.04  (*SD* = 0.95) | Total = 56  Mean = 2.07  (*SD* = 0.87) |
| *Hits* | Total = 26  Mean = 1.30  (*SD* = 0.57) | Total = 46  Mean = 1.84  (*SD* = 1.60) | Total = 43  Mean = 1.79  (*SD* = 1.02) | Total = 49  Mean = 1.81  (*SD* = 0.92) |
| *Percentage* | 81.25% | 79.31% | 87.76% | 87.50% |
| *Ranking* | None: 2 (10.00%)  First: 13 (65.00%)  Second: 5 (25.00%) | None: 5 (20.00%)  First: 15 (60.00%)  Second: 5 (20.00%) | None: 3 (12.50%)  First: 16 (66.66%)  Second: 5 (20.84%) | None: 1 (3.71%)  First: 22 (81.48%)  Second: 4 (14.81%) |

We were interested in whether both the control and intervention group differed in these shooting characteristics in the pre- and post-test. However, for *Shots* and *Hits*, a repeated measures ANOVA (*Time* × *Group*) showed neither significant main effects nor significant interaction effects. For *Ranking*, a two-sided Fisher-test showed that both groups did not differ significantly in the pre-test (*p* = 0.760) nor in the post-test (*p* = 0.446). Moreover, we checked for an effect of the number of *Lessons* and the self-estimated handgun *Proficiency* on the sample’s performance. Although we observed p-values <0.05 for both covariates, the practical influence tended towards 0.
